# 3-Cyano­anilinium bromide

**DOI:** 10.1107/S1600536809034941

**Published:** 2009-09-09

**Authors:** Bo Wang

**Affiliations:** a Ordered Matter Science Research Center, College of Chemistry and Chemical Engineering, Southeast University, Nanjing 210096, People’s Republic of China

## Abstract

In the cation of the title compound, C_7_H_7_N_2_^+^·Br^−^, all non-H atoms are essentially coplanar [r.m.s. deviation = 0.010 (5) Å]. The compound is isomorphous with the chloride analogue. In the crystal, the cations and anions are connected by N—H⋯Br hydrogen bonds.

## Related literature

For applications of metal-organic coordination compounds, see: Fu *et al.* (2007 ▶); Chen *et al.* (2001 ▶); Fu & Xiong (2008 ▶); Xiong *et al.* (1999 ▶); Xie *et al.* (2003 ▶); Zhao *et al.* (2004 ▶). For nitrile derivatives, see: Fu *et al.* (2008 ▶); Wang *et al.* 2002 ▶. For the chloride analogue, see: Wen (2008 ▶).
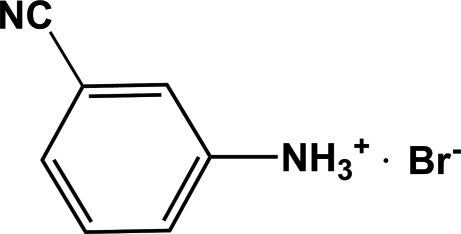


## Experimental

### 

#### Crystal data

C_7_H_7_N_2_^+^·Br^−^
*M* *_r_* = 199.06
Triclinic, 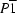

*a* = 4.6396 (9) Å
*b* = 6.1757 (12) Å
*c* = 13.542 (3) Å
α = 93.07 (3)°
β = 96.22 (3)°
γ = 97.33 (3)°
*V* = 381.68 (13) Å^3^
*Z* = 2
Mo *K*α radiation
μ = 5.31 mm^−1^
*T* = 298 K
0.40 × 0.05 × 0.05 mm

#### Data collection

Rigaku Mercury2 diffractometer
Absorption correction: multi-scan (*CrystalClear*; Rigaku, 2005 ▶) *T*_min_ = 0.90, *T*_max_ = 1.00
3777 measured reflections
1716 independent reflections
1378 reflections with *I* > 2σ(*I*)
*R*_int_ = 0.063

#### Refinement

*R*[*F* ^2^ > 2σ(*F* ^2^)] = 0.052
*wR*(*F* ^2^) = 0.134
*S* = 1.10
1716 reflections
92 parameters
H-atom parameters constrained
Δρ_max_ = 0.71 e Å^−3^
Δρ_min_ = −0.75 e Å^−3^

### 

Data collection: *CrystalClear* (Rigaku, 2005 ▶); cell refinement: *CrystalClear*; data reduction: *CrystalClear*; program(s) used to solve structure: *SHELXS97* (Sheldrick, 2008 ▶); program(s) used to refine structure: *SHELXL97* (Sheldrick, 2008 ▶); molecular graphics: *SHELXTL* (Sheldrick, 2008 ▶); software used to prepare material for publication: *SHELXTL*.

## Supplementary Material

Crystal structure: contains datablocks I, global. DOI: 10.1107/S1600536809034941/bx2236sup1.cif

Structure factors: contains datablocks I. DOI: 10.1107/S1600536809034941/bx2236Isup2.hkl

Additional supplementary materials:  crystallographic information; 3D view; checkCIF report

## Figures and Tables

**Table 1:** Hydrogen-bond geometry (Å, °)

*D*—H⋯*A*	*D*—H	H⋯*A*	*D*⋯*A*	*D*—H⋯*A*
N2—H2*A*⋯Br1^i^	0.89	2.59	3.434 (4)	159
N2—H2*B*⋯Br1^ii^	0.89	2.46	3.337 (4)	169
N2—H2*C*⋯Br1	0.89	2.45	3.299 (4)	160

Symmetry codes: (i) 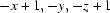; (ii) 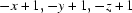.

**Fig. 1.**
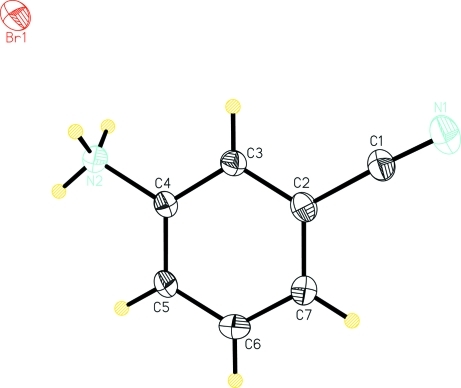
A view of the title compound with the atomic numbering scheme. Displacement ellipsoids are drawn at the 30% probability level.

**Fig. 2.**
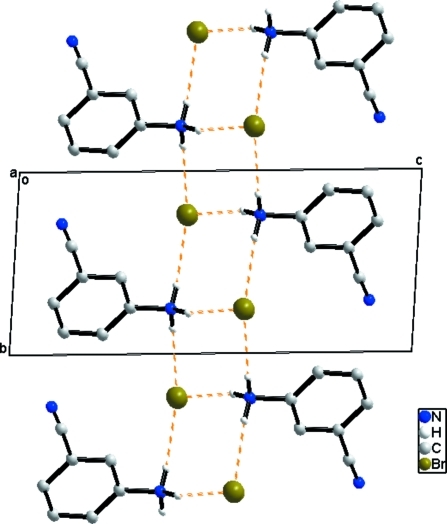
The crystal packing of the title compound, viewed along the a axis showing the N—H···Br interactions (dotted line) in the title compound. H atoms not involved in hydrogen bonding (dashed lines) have been omitted for clarity.

## supplementary crystallographic information

### Comment

The construction of metal-organic coordination compounds has attracted much attention owing to potential functions, such as permittivity, fluorescence, magnetism and optical properties (Fu *et al.*, 2007; Chen *et al.*, 2001; Fu & Xiong (2008); Xie *et al.*, 2003; Zhao *et al.*,2004; Xiong *et al.*, 1999). Nitrile derivatives are a class of excellent ligands for the construction of novel metal-organic frameworks. (Wang *et al.* 2002; Fu *et al.*, 2008). We report here the crystal structure of the title compound, which is isomorphous with the chloride analogue (Wen, 2008). In the cation all non-H atoms are essentially coplanar [r.m.s. deviation 0.010 (5) Å]. In the crystal structure, the organic cations and bromide ions are connected by  N—H···Br hydrogen bonds along *b* axis, (Table 1), (Fig. 2).

### Experimental

The commercial 3-aminobenzonitrile (3 mmol, 0.55 g) and HBr (0.5 ml) were dissolved in ethanol (20 ml). Colourless block-shaped crystals of the title compound suitable for X-ray analysis were obtained by slow evaporation at room temperature.

### Refinement

All H atoms attached to C and N atoms were positioned geometrically and treated as riding, with C-H = 0.93 Å, N-H = 0.89 Å and *U*_iso_(H) = 1.2U_eq_(C) and *U*_iso_(H) = 1.5U_eq_(N). A rotating-group model was used for the -NH_3_ group.

### Crystal data

**Table:**

C_7_H_7_N_2_^+^·Br^−^	*Z* = 2
*M**_r_* = 199.06	*F*(000) = 196
Triclinic, *P*1	*D*_x_ = 1.732 Mg m^−^^3^
Hall symbol: -P 1	Mo *K*α radiation, λ = 0.71073 Å
*a* = 4.6396 (9) Å	Cell parameters from 1378 reflections
*b* = 6.1757 (12) Å	θ = 3.0–27.5°
*c* = 13.542 (3) Å	µ = 5.31 mm^−^^1^
α = 93.07 (3)°	*T* = 298 K
β = 96.22 (3)°	Block, colourless
γ = 97.33 (3)°	0.40 × 0.05 × 0.05 mm
*V* = 381.68 (13) Å^3^	

### Data collection

**Table:**

Rigaku Mercury2 diffractometer	1716 independent reflections
Radiation source: fine-focus sealed tube	1378 reflections with *I* > 2σ(*I*)
graphite	*R*_int_ = 0.063
Detector resolution: 13.6612 pixels mm^-1^	θ_max_ = 27.5°, θ_min_ = 3.0°
CCD profile fitting scans	*h* = −6→5
Absorption correction: multi-scan (*CrystalClear*; Rigaku, 2005)	*k* = −8→8
*T*_min_ = 0.90, *T*_max_ = 1.00	*l* = −17→17
3777 measured reflections	

### Refinement

**Table:**

Refinement on *F*^2^	Primary atom site location: structure-invariant direct methods
Least-squares matrix: full	Secondary atom site location: difference Fourier map
*R*[*F*^2^ > 2σ(*F*^2^)] = 0.052	Hydrogen site location: inferred from neighbouring sites
*wR*(*F*^2^) = 0.134	H-atom parameters constrained
*S* = 1.10	*w* = 1/[σ^2^(*F*_o_^2^) + (0.053*P*)^2^ + 0.0394*P*] where *P* = (*F*_o_^2^ + 2*F*_c_^2^)/3
1716 reflections	(Δ/σ)_max_ < 0.001
92 parameters	Δρ_max_ = 0.71 e Å^−^^3^
0 restraints	Δρ_min_ = −0.75 e Å^−^^3^

### Special details

**Table:**

Geometry. All esds (except the esd in the dihedral angle between two l.s. planes) are estimated using the full covariance matrix. The cell esds are taken into account individually in the estimation of esds in distances, angles and torsion angles; correlations between esds in cell parameters are only used when they are defined by crystal symmetry. An approximate (isotropic) treatment of cell esds is used for estimating esds involving l.s. planes.
Refinement. Refinement of F^2^ against ALL reflections. The weighted R-factor wR and goodness of fit S are based on F^2^, conventional R-factors R are based on F, with F set to zero for negative F^2^. The threshold expression of F^2^ > 2sigma(F^2^) is used only for calculating R-factors(gt) etc. and is not relevant to the choice of reflections for refinement. R-factors based on F^2^ are statistically about twice as large as those based on F, and R- factors based on ALL data will be even larger.

### Fractional atomic coordinates and isotropic or equivalent isotropic displacement parameters (Å^2^)

**Table:**

	*x*	*y*	*z*	*U*_iso_*/*U*_eq_	
N2	0.6256 (9)	0.2461 (6)	0.6051 (3)	0.0419 (10)	
H2A	0.7437	0.1446	0.5990	0.063*	
H2B	0.7175	0.3755	0.5927	0.063*	
H2C	0.4653	0.2118	0.5619	0.063*	
N1	−0.0113 (11)	0.7158 (8)	0.8871 (4)	0.0575 (13)	
C4	0.5438 (10)	0.2565 (7)	0.7064 (4)	0.0339 (10)	
C3	0.3783 (10)	0.4177 (7)	0.7330 (3)	0.0349 (10)	
H3	0.3215	0.5166	0.6877	0.042*	
C2	0.3001 (10)	0.4273 (7)	0.8286 (4)	0.0350 (10)	
C5	0.6331 (11)	0.1113 (7)	0.7719 (4)	0.0389 (11)	
H5	0.7446	0.0048	0.7527	0.047*	
C7	0.3878 (12)	0.2814 (8)	0.8962 (4)	0.0429 (12)	
H7	0.3345	0.2889	0.9604	0.051*	
C6	0.5540 (12)	0.1259 (8)	0.8677 (4)	0.0473 (13)	
H6	0.6146	0.0287	0.9132	0.057*	
C1	0.1216 (11)	0.5910 (8)	0.8593 (4)	0.0422 (12)	
Br1	0.09268 (11)	0.23870 (7)	0.42210 (4)	0.0448 (2)	

### Atomic displacement parameters (Å^2^)

**Table:**

	*U*^11^	*U*^22^	*U*^33^	*U*^12^	*U*^13^	*U*^23^
N2	0.053 (3)	0.044 (2)	0.032 (2)	0.0176 (19)	0.0038 (19)	0.0003 (18)
N1	0.058 (3)	0.052 (3)	0.067 (3)	0.020 (2)	0.016 (3)	−0.004 (2)
C4	0.040 (3)	0.028 (2)	0.033 (3)	0.0073 (18)	0.004 (2)	−0.0017 (18)
C3	0.039 (3)	0.033 (2)	0.032 (3)	0.010 (2)	0.000 (2)	0.0010 (19)
C2	0.030 (2)	0.034 (2)	0.041 (3)	0.0057 (18)	0.005 (2)	−0.002 (2)
C5	0.049 (3)	0.034 (2)	0.037 (3)	0.018 (2)	0.007 (2)	−0.001 (2)
C7	0.056 (3)	0.039 (3)	0.035 (3)	0.011 (2)	0.010 (2)	0.000 (2)
C6	0.062 (4)	0.040 (3)	0.045 (3)	0.015 (2)	0.010 (3)	0.014 (2)
C1	0.045 (3)	0.040 (3)	0.044 (3)	0.012 (2)	0.011 (2)	0.000 (2)
Br1	0.0573 (4)	0.0399 (3)	0.0423 (4)	0.0214 (2)	0.0112 (3)	0.0037 (2)

### Geometric parameters (Å, °)

**Table:**

N2—C4	1.463 (6)	C3—H3	0.9300
N2—H2A	0.8900	C2—C7	1.384 (7)
N2—H2B	0.8900	C2—C1	1.457 (6)
N2—H2C	0.8900	C5—C6	1.388 (7)
N1—C1	1.121 (6)	C5—H5	0.9300
C4—C5	1.365 (6)	C7—C6	1.371 (7)
C4—C3	1.388 (6)	C7—H7	0.9300
C3—C2	1.383 (7)	C6—H6	0.9300
			
C4—N2—H2A	109.5	C3—C2—C1	120.2 (4)
C4—N2—H2B	109.5	C7—C2—C1	119.1 (5)
H2A—N2—H2B	109.5	C4—C5—C6	118.6 (4)
C4—N2—H2C	109.5	C4—C5—H5	120.7
H2A—N2—H2C	109.5	C6—C5—H5	120.7
H2B—N2—H2C	109.5	C6—C7—C2	119.4 (5)
C5—C4—C3	122.0 (4)	C6—C7—H7	120.3
C5—C4—N2	119.8 (4)	C2—C7—H7	120.3
C3—C4—N2	118.2 (4)	C7—C6—C5	121.0 (5)
C2—C3—C4	118.2 (4)	C7—C6—H6	119.5
C2—C3—H3	120.9	C5—C6—H6	119.5
C4—C3—H3	120.9	N1—C1—C2	177.0 (6)
C3—C2—C7	120.7 (4)		
			
C5—C4—C3—C2	−0.9 (7)	N2—C4—C5—C6	179.6 (5)
N2—C4—C3—C2	179.8 (4)	C3—C2—C7—C6	0.0 (7)
C4—C3—C2—C7	0.7 (7)	C1—C2—C7—C6	179.5 (5)
C4—C3—C2—C1	−178.8 (4)	C2—C7—C6—C5	−0.6 (8)
C3—C4—C5—C6	0.3 (7)	C4—C5—C6—C7	0.5 (8)

### Hydrogen-bond geometry (Å, °)

**Table:**

*D*—H···*A*	*D*—H	H···*A*	*D*···*A*	*D*—H···*A*
N2—H2A···Br1^i^	0.89	2.59	3.434 (4)	159
N2—H2B···Br1^ii^	0.89	2.46	3.337 (4)	169
N2—H2C···Br1	0.89	2.45	3.299 (4)	160

Symmetry codes: (i) −*x*+1, −*y*, −*z*+1; (ii) −*x*+1, −*y*+1, −*z*+1.
